# GeoBoreNet: RGB–Geometry Fusion for Cross-Scene Borehole Detection

**DOI:** 10.3390/s26185963

**Published:** 2026-09-20

**Authors:** Xuesong Liu, Wenbo Cao, Anke Xu, Emmett J. Ientilucci

**Affiliations:** 1 Chester F. Carlson Center for Imaging Science, Rochester Institute of Technology, Rochester, NY 14623, USA; xl2088@rit.edu; 2 Independent Researcher, Washington, DC 20005, USA; 3 Independent Researcher, New York, NY 10001, USA

**Keywords:** borehole detection, textured 3D reconstruction, RGB–geometry fusion, cross-scene evaluation

## Abstract

Borehole detection in textured quarry reconstructions is challenging because shadows, rocks, and fractured ground can resemble visible surface openings. We introduce GeoBoreNet, which augments RGB with four pixel-aligned inputs derived from reconstructed surface height: local residual relief, spatial gradient magnitude, spatial Laplacian response, and mesh support. Plane removal and resolution-adapted local-background subtraction encode surface morphology, and an expanded first convolution incorporates these channels into standard detectors. Experiments evaluate DINO-R50 and YOLO11x across 11 source scenes using scene-disjoint leave-one-scene-out testing, with five paired training seeds for DINO-R50. In pooled cross-fold evaluation, adding geometry increases AP from 0.483 to 0.548 for DINO-R50 and from 0.385 to 0.450 for YOLO11x, an absolute gain of 0.065 for each detector. Component and local-background scale studies further assess the geometry representation. DINO-R50 source-object-unique AP increases from 0.448 to 0.493 at cross-patch NMS IoU 0.4. In evaluated search regions containing zero-object tiles, AP increases from 0.380 to 0.430 for DINO-R50 and from 0.300 to 0.360 for YOLO11x. These results show that local surface morphology complements RGB for two-dimensional borehole detection in the evaluated scenes.

## 1. Introduction

**Figure 1:**
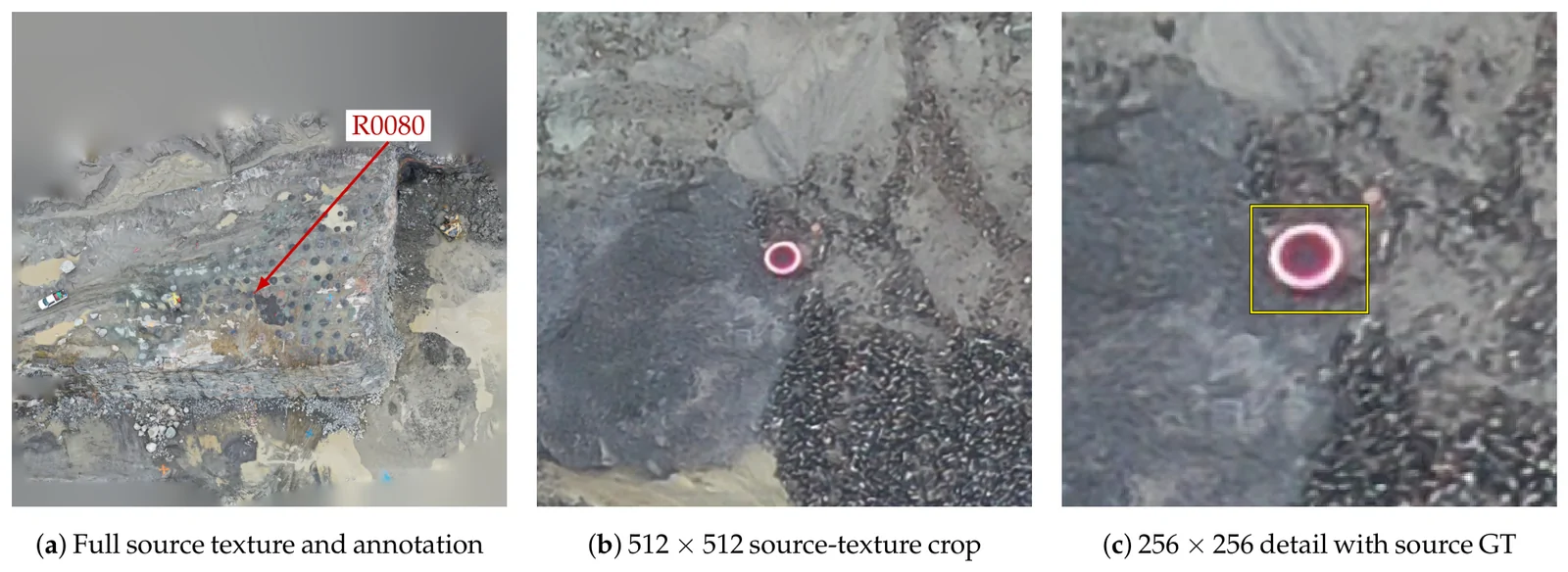
Borehole annotation example (S02, R0080). (**a**) Complete 20,480 × 20,480 source texture, with a callout locating the annotation. (**b**) A 512×512 crop centered on the same opening. (**c**) The central 256×256 region of (**b**), enlarged to show the original 60×55-pixel ground-truth rectangle (yellow) around the visible opening and collar. Both crops use native texture coordinates, before mesh/UV projection.

Borehole detection supports drill-pattern verification, blast monitoring, and automated mine inspection [1,2,3]. Here, a borehole is defined as the visible surface opening to be localized by a two-dimensional bounding box. Textured three-dimensional (3D) quarry reconstructions provide broad surface coverage, but openings occupy only a small part of each detector crop and can resemble shadows, dark rocks, tire marks, or fractured ground. Variation in these backgrounds makes transfer to a new reconstruction a central challenge. Separating source scenes during evaluation helps assess performance beyond scene-specific appearance and limits the effects of spatial dependence between training and test samples [4]. Figure 1 shows a source annotation in the full texture and progressively enlarged local views.

Traditional borehole-localization pipelines use hand-designed image descriptors with classifiers or fit geometric templates to range data [3,5]. These methods make useful appearance and shape cues explicit, but their assumptions can become restrictive when illumination, ground texture, viewing direction, or aperture visibility changes.

Deep-learning methods replace fixed descriptors with learned features and have been applied to RGB-D segmentation, tunnel-face detection, and binocular localization [6,7,8]. For RGB-only detection, dark background structures can still resemble openings even when the detector learns more expressive features. Aligned mesh height supplies local relief measurements that RGB texture does not directly provide, giving detectors an additional cue for separating openings from background structures. Two authors previously studied RGB-only contextual learning with YOLO-family detectors [9]; the present study extends the evaluation to RGB–geometry inputs across detector families and held-out source scenes.

GeoBoreNet combines RGB with four channels derived from the reconstructed surface. Plane removal and local-background subtraction reduce coordinate offsets and broad surface slopes, producing a residual relief map with positive values for depressions. This map, its spatial gradient magnitude and Laplacian response, and a mesh-support mask are aligned with RGB. These quantities are computed from the surface raster before inference and enter the detector through an expanded seven-channel first convolution. Paired experiments with DINO-R50 and YOLO11x assess the representation in transformer and one-stage detectors, complemented by comparisons with Deformable DETR, Conditional DETR, and Cascade R-CNN. Our main contributions are:

- **A locally referenced RGB–geometry representation.** GeoBoreNet encodes surface relief, spatial derivatives, and mesh support as four RGB-aligned channels. A seven-channel first convolution incorporates these inputs while preserving the rest of the detector architecture and its loss functions.
- **Controlled comparisons across scenes and detectors.** Eleven scene-disjoint folds, five paired DINO-R50 seeds, and comparisons across five detector families assess transfer and training variability. Support-mask ablations and local-background scale controls examine the effects of the geometry inputs and their preprocessing.
- **Evaluation of repeated targets, background, and missing geometry.** Source-object-unique aggregation addresses repeated-target weighting, search regions containing zero-object tiles quantify false-positive burden, and geometry-dropout experiments assess performance with and without geometry at inference.

## 2. Related Work

### 2.1. Traditional Feature- and Geometry-Based Localization

Feature-based localization identifies boreholes through local intensity or shape patterns. Valencia et al. [5] applied histogram-of-oriented-gradient descriptors and a support vector machine to sliding windows from UAV orthomosaics and digital elevation models (DEMs). Training included positive and negative windows, while non-maximum suppression consolidated repeated detections around individual holes. This approach links photogrammetric survey products to as-drilled locations, but makes the window scale and the discriminative quality of local gradients central to detection. Similar boundaries in fractured rock, shadows, and hole rims can complicate classification, especially when the aperture occupies only a small part of the window.

Range-based methods instead constrain localization through explicit surface geometry. Liu et al. [3] extracted the raised cone of drill waste from LiDAR, projected its points into virtual depth images, and fitted circles to guide robotic sensor insertion. Their pipeline filters shallow pits that could otherwise be mistaken for holes. Such geometric constraints reduce dependence on texture, but require an identifiable cone and sufficient sampling of the opening. Weak relief, incomplete collars, and sparse reconstruction can make those conditions harder to satisfy. The applicability of these methods therefore depends on both the visibility of the aperture boundary and the sensing configuration.

### 2.2. Learning-Based Blasthole Detection

Learned feature enhancement has addressed several sources of weak visual evidence. Pan et al. [7] coupled multiscale CycleGAN features with YOLOv7 to improve contrast and suppress over-exposure in roadway-face images. Zhang et al. [10] combined top-down feature fusion, staged training, and false-detection filtering in Faster R-CNN to recognize tunnel-face blastholes and estimate their counts and spacing. For far-view quarry imagery, Liu et al. [9] introduced adaptive augmentation, embedding stabilization, and contextual refinement. These approaches strengthen contrast, preserve small-target features, or incorporate surrounding context, while remaining dependent on the visual distinction between apertures and background structures.

Depth contributes through both recognition and subsequent positioning. Zhang et al. [6] developed adaptive RGB-D fusion for semantic segmentation, using pseudo-labeling and self-training to reduce manual labeling requirements. Yi et al. [11] detected holes with YOLOv8 and used camera depth to estimate filling positions. Hu et al. [8] combined attention and multiscale improvements to YOLOv10 with binocular 3D localization. These uses of geometry serve different purposes: fusing depth features can affect foreground discrimination, whereas recovering depth after image detection supplies metric coordinates for an already identified target. Improvements in positioning accuracy consequently do not, by themselves, establish improved detection of visually ambiguous openings.

Surface elevation also affects transfer between survey products. Valencia et al. [5] trained CNN classifiers on orthomosaic or DEM patches and included held-out datasets in their DEM experiments. Normalizing elevation improved performance when absolute elevations differed, highlighting the influence of representation on transfer. The image and DEM classifiers were evaluated separately, leaving their complementarity within a joint detector unresolved. Evaluation protocols also affect the interpretation of reported gains. Randomly sampled images, as used by Pan et al. [7], and held-out surveys expose models to different degrees of scene variation. Spatial dependence can make random splits underestimate prediction error [4], particularly when nearby or overlapping crops share targets and background.

### 2.3. Small-Target Features and Context

The limited spatial extent of an aperture connects borehole detection to small-object detection in aerial imagery. Ni et al. [12] augmented YOLOv8s with parallel multiscale feature extraction and a feature-compensation pyramid, combining receptive fields of different sizes to retain detail and context. In transformer detectors, Deformable DETR restricts attention to sparse sampling locations around reference points, improving convergence and small-object detection [13]. DINO further improves query initialization and box prediction through contrastive denoising and iterative refinement [14]. These developments address feature resolution, scale interaction, and optimization. Context can strengthen a weak target signal, but can also encode background patterns specific to the training scenes; architectural improvements and transfer to new backgrounds therefore require distinct evaluation.

### 2.4. Geometry Encoding, Fusion, and Missing Inputs

Geometry can provide complementary structure, but its encoding determines which information is available to a model. Gupta et al. [15] represented depth through disparity, height above ground, and angle with gravity, obtaining stronger learned features than with raw depth. Hesse’s local relief models [16] remove broad terrain forms to expose small elevation differences. Both approaches express geometry relative to a spatial reference. For low-relief targets, this distinction matters because absolute height can vary independently of object identity, while the choice of reference scale determines which local structures remain visible.

The fusion stage introduces a separate design trade-off. Audebert et al. [17] compared early and late fusion for urban semantic labeling: early fusion supported joint feature learning, but was more sensitive to missing data. Ophoff et al. [18] varied the fusion location throughout a YOLOv2 detector and showed that detection performance depends on where RGB and depth are combined. Alignment and additional channels alone are also insufficient. Zambanini et al. [19] found that adding a stereo-derived surface model did not improve parking-car detection because reconstruction quality was inadequate at the target scale. This result distinguishes geometric coverage from the availability of discriminative shape information.

Training with incomplete inputs addresses the dependence of fusion models on modality availability. ModDrop randomly removes modality streams during training to support prediction when some inputs are missing [20]. Mena et al. [21] combined sensor dropout with mutual distillation for crop and tree classification under missing sensors. These studies treat complete-input accuracy and missing-input robustness as separate properties: a gain from joint observations does not guarantee that the same model remains effective when one modality is unavailable.

The present study evaluates locally referenced mesh geometry as an additional input for borehole detection. Paired RGB and RGB–geometry comparisons in DINO-R50 and YOLO11x [22] assess its contribution across held-out source scenes, while separate experiments examine performance when geometry is absent.

## 3. Materials and Methods

### 3.1. Problem Formulation

Each sample consists of an RGB texture patch $I\in R^{H\times W\times 3}$, a pixel-aligned mesh-height raster $G\in R^{H\times W}$, a binary geometry-support raster *v*, and a set of axis-aligned borehole boxes Y. A deterministic transform ψ converts (G,v) to four channels $X_{G}\in R^{H\times W\times 4}$. The channels are stacked with RGB and passed to a detector $D_{\omega }$:

(1) $$\hat{Y}=D_{\omega }\;\left({\mathrm{concat}}_{\mathrm{ch}}\left(I,X_{G}\right)\right).$$

**Figure 2:**
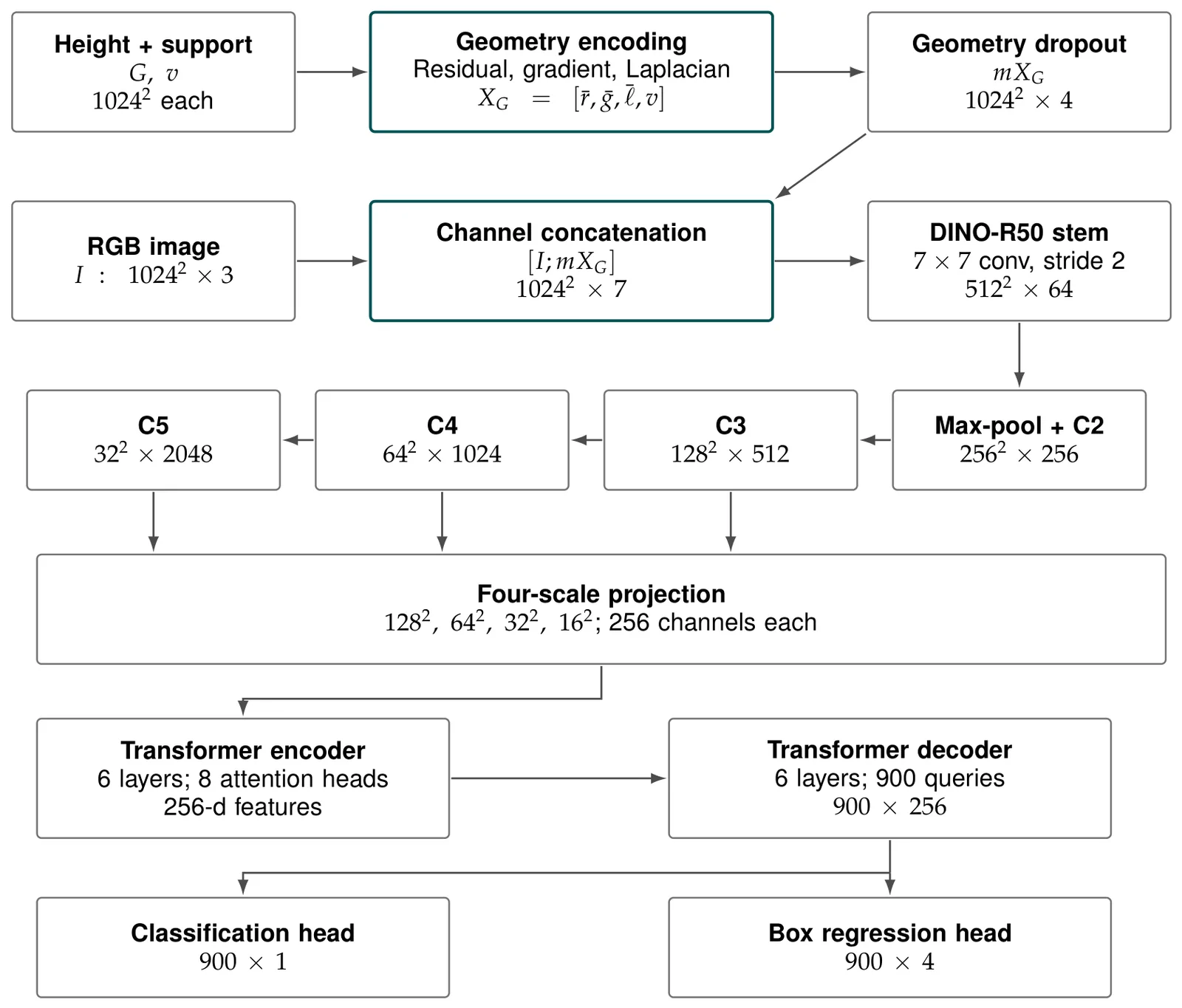
GeoBoreNet input preparation and DINO-R50 architecture. Geometry encoding removes the fitted plane and local background, computes spatial derivatives, and normalizes the continuous channels. Encoding and seven-channel input construction are shared with YOLO11x. C2–C5 are ResNet-50 stages; Table 1 gives normalization, activation, and loss settings. Shapes are height × width × channels, with $n^{2}=n\times n$. One mask *m* applies jointly to all four geometry channels; m=1 for the main comparison.

The experiments compare RGB-only, derived-geometry-only, and RGB+Derived inputs with matched detector settings apart from the corresponding first-convolution input width. Geometry dropout modifies only the geometry portion of the seven-channel input, as defined in Section 3.7. Figure 2 and Table 1 summarize the input preparation and DINO-R50 architecture.

### 3.2. Dataset Construction and Scene-Level Splits

The source data consist of textured triangular surface meshes: OBJ files store XYZ vertices and UV coordinates, MTL files link the materials, and JPEG files provide the RGB textures. The experimental subset retains 11 annotated source scenes; byte-identical aliases in the broader delivery are not treated as additional scenes. Native texture dimensions vary by scene. Table 2 summarizes the aligned corpus. Table 3 reports detector-grid resolutions from the preprocessing metadata. Texture coordinates are mapped to this grid through mesh UV coordinates. Mesh *X* and *Y* define the top-down raster plane, and *Z* is height. Raster spacing $\rho_{s}$ follows the mesh coordinate system and is recorded in meters per pixel in the reconstruction metadata. Within each raster cell, height is obtained by barycentric interpolation of candidate triangles, retaining the maximum-*Z* fragment where projections overlap. The binary raster *v* records mesh support. RGB and height products are then sampled on a common detector grid, with corresponding pixels sharing the same raster indices. An audit of 195 source–patch–source coordinate round trips found a maximum absolute error of 0.0 pixels.

The source annotations are two-point rectangles stored in LabelMe-compatible JSON. Two scene-specific legacy label strings were reviewed against their image regions and normalized to the single detection class borehole; the original strings remain unchanged in the source files. The inventory contains 683 texture-coordinate rectangles, of which 675 have a valid projection to the detector grid. The other eight remain in the source inventory but do not generate detector samples. The number of source annotations per scene ranges from 18 to 169, and the median source-coordinate box width and height are 52.5 and 50.0 texture pixels.

**Figure 3:**
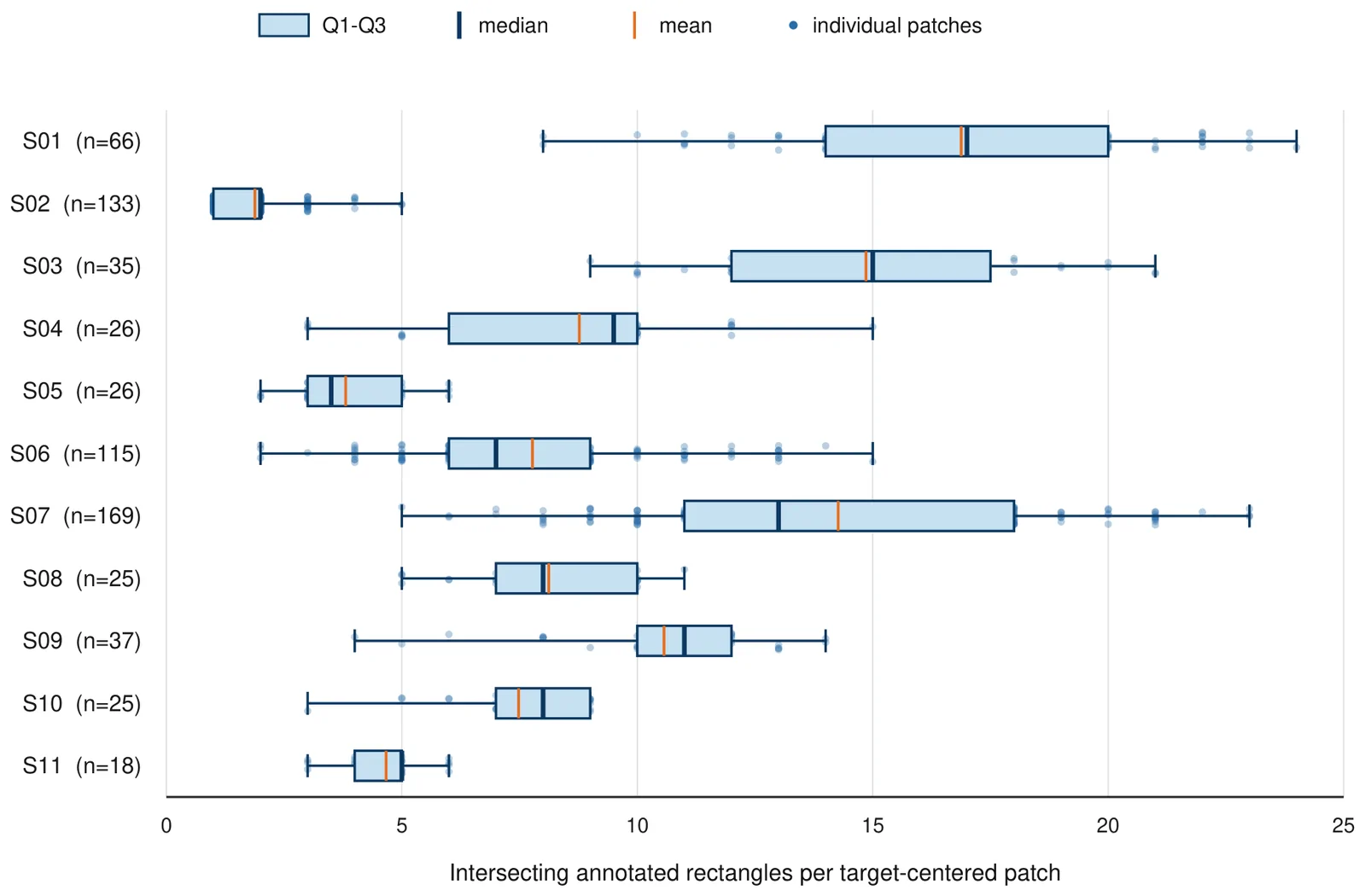
Distribution of positive-area annotated-rectangle intersections per 1024×1024 target-centered patch for all 675 constructed patches. Boxes span Q1–Q3 computed by linear interpolation, dark-blue lines mark medians, thin orange lines spanning the box height mark means, whiskers show the observed minima and maxima, and points show individual patches with vertical jitter for visibility. These counts correspond to the 6381 pre-conversion intersections; after seven one-pixel boundary slivers become degenerate under the detector-manifest convention, 6374 box records remain for evaluation.

Target-centered construction creates fixed-size, aligned RGB and geometry inputs around sparse source annotations for the representation comparison. For every projectable rectangle, one 1024×1024 patch is centered on that rectangle; consequently, patches are not generated on a fixed-stride grid, and this construction produces no negative patches. A patch retains every projected rectangle with a strictly positive-area intersection and clips it to the patch boundary without a minimum visible-fraction threshold. This rule gives 6381 positive-area patch–rectangle intersections. Conversion to the detector manifest uses the inclusive pixel range [0,1023]; seven one-pixel boundary slivers become degenerate under that convention and are excluded, leaving 6374 evaluated box records. Windows may extend beyond raster support, where v=0. Because neighboring targets can enter several overlapping patches, these records are repeated observations of the 675 projectable source rectangles rather than distinct physical boreholes. Of the 675 rectangles, 615 occur in more than one patch; the multiplicity has minimum, median, and maximum values of 1, 9, and 25. Table 2 and Table 3 summarize the aligned corpus, and Figure 3 shows the full per-patch count distributions.

Patch-level target counts differ substantially across scenes (Figure 3). The median is 2 in S02 and 17 in S01; S07 spans 5–23 intersections per patch, with the middle 50% between 11 and 18. These distributions describe occupancy within fixed-size detector windows; comparisons across scenes must account for raster resolution and overlapping patch construction.

The 675 patches form the target-centered corpus. The operational evaluation in Section 4.5 uses a separate tile set containing both positive and zero-object tiles. Each patch inherits the source-scene identifier of its parent texture. In each of 11 outer folds, one complete source scene is reserved for testing, the next scene in the cyclic S01–S11 ordering is used for validation and checkpoint selection, and the other nine scenes are used for training. Training retains the target-centered patches and their repeated target observations from all nine training scenes. All patches from a source share the same role within a fold. This grouping prevents crops from one delivered reconstruction from crossing training, validation, and test roles and evaluates transfer between source scenes. Because the acquisition records do not establish that every source represents a distinct physical quarry, the claims are stated at the source-scene level.

### 3.3. Annotation Criteria, Projection, and Quality Control

The detection class is the visible surface opening of a borehole. Four trained annotators separately reviewed and annotated the same set of full-resolution texture scenes. Their task training covered representative boreholes and common visual confusers, including shadows, rocks, fractures, and isolated dark texture regions. A candidate was treated as a borehole when a discernible, approximately circular or elliptical surface aperture was visible, optionally with a rim or collar. Regular drill-pattern spacing was used as supporting scene context, but was not sufficient by itself to assign the class.

Annotators first identified the visual center of the aperture and then adjusted a tight axis-aligned rectangle to enclose the complete visible opening or collar. Cast shadows, surrounding fractured ground, and loose material were excluded whenever they could be distinguished from the aperture. The four annotation sets were consolidated by a fifth researcher familiar with both object-detection algorithms and blasting practice. This adjudicator reviewed presence/absence disagreements, inspected ambiguous candidates in their full-scene context, and adjusted the final rectangles. Candidates that remained indistinguishable from background confusers after adjudication were excluded. The final two-point rectangles were stored in LabelMe-compatible JSON. Legacy labels are mapped to the single detector class without modifying the original annotation files.

**Figure 4:**
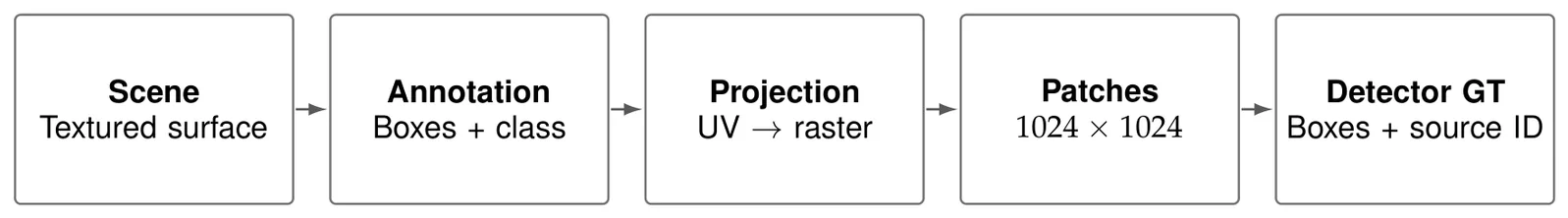
Annotation and data preparation. Source rectangles are projected from texture coordinates to the detector raster and clipped to target-centered patches. Each detector box retains its source-object ID and scene assignment. Figure 1 shows a source annotation in S02.

Quality control is performed at four levels. First, independent annotation by four trained annotators and adjudication by a fifth researcher provide consensus and domain-informed review. Second, source records retain the original file, label, rectangle index, and disposition. Third, source-to-raster and raster-to-patch coordinate transforms are checked by round-trip tests and by overlaying representative rectangles on the aligned RGB and geometry products. Fourth, the generated detector manifests are audited for class mapping, degenerate boxes, repeated source-object identifiers, and scene-disjoint fold assignment. Truncated rectangles are clipped only after projection to a patch, and projected boxes with zero area are excluded from detector evaluation while remaining traceable to their source record. Figure 4 summarizes this sequence.

### 3.4. Plane Detrending and Local Depression Residual

Let $z_{ij}$ be the mesh-derived height at row *i* and column *j*. We use centered pixel coordinates $x_{j}=j-\left(W-1\right)/2$ and $y_{i}=i-\left(H-1\right)/2$. Height offsets and broad surface slopes introduce variation unrelated to local borehole shape. Let $\Omega =\{\left(i,j\right):v_{ij}=1\}$ denote the supported pixels. Unsupported height cells are filled for filtering by 20 iterations of four-neighbor diffusion; they remain identified by v=0. We fit a plane by least squares to a stride-8 sample $\Omega_{s}\subset \Omega$:

(2) $$\left(a,b,c\right)=\arg\min_{a,b,c}\sum_{\left(i,j\right)\in \Omega_{s}}{\left[z_{ij}-\left(ax_{j}+by_{i}+c\right)\right]}^{2},$$

and define the plane-detrended field

(3) $$z_{ij}^{p}=z_{ij}-\left(ax_{j}+by_{i}+c\right).$$

A uniform box operator $B_{k_{s}}$, evaluated with edge padding, then estimates the local background for scene *s*, and the encoded depression residual is

(4) $$r_{ij}=B_{k_{s}}{\left(z^{p}\right)}_{ij}-z_{ij}^{p}.$$

Let *L* denote the local-background span in the same length unit as the recorded raster resolution $\rho_{s}$. The reference setting is nominally L=0.30 m. Its scene-specific odd pixel window is

(5) $$q_{s}=\mathrm{round}\;\left(\frac{L}{\rho_{s}}\right),\;k_{s}=\max\;\left(3,2\left\lfloor \frac{q_{s}}{2}\right\rfloor +1\right).$$

Thus, an even rounded value is increased by one and an odd value is retained, with a minimum of three pixels. For the reference setting, $k_{s}$ ranges from 3 to 61 pixels. This rule adapts the neighborhood to the recorded grid resolution, although integer rounding and the minimum window limit scale equivalence at coarse resolutions. The local window separates a depression from the surrounding surface trend. Its reference span of 0.30 m sets the background neighborhood; points below this background have positive residuals and elevations above it have negative residuals. Section 4.2.3 evaluates half and twice the reference span.

### 3.5. Morphology-Aware Geometry Encoding

Boreholes can produce localized surface depressions. From the signed local residual *r* in Equation (4), we compute a discrete gradient magnitude

(6) $$g_{ij}=\sqrt{{\left(\frac{r_{i,j+1}-r_{i,j-1}}{2}\right)}^{2}+{\left(\frac{r_{i+1,j}-r_{i-1,j}}{2}\right)}^{2}}$$

and a four-neighbor discrete Laplacian response

(7) $$l_{ij}=r_{i-1,j}+r_{i+1,j}+r_{i,j-1}+r_{i,j+1}-4r_{ij}.$$

**Figure 5:**
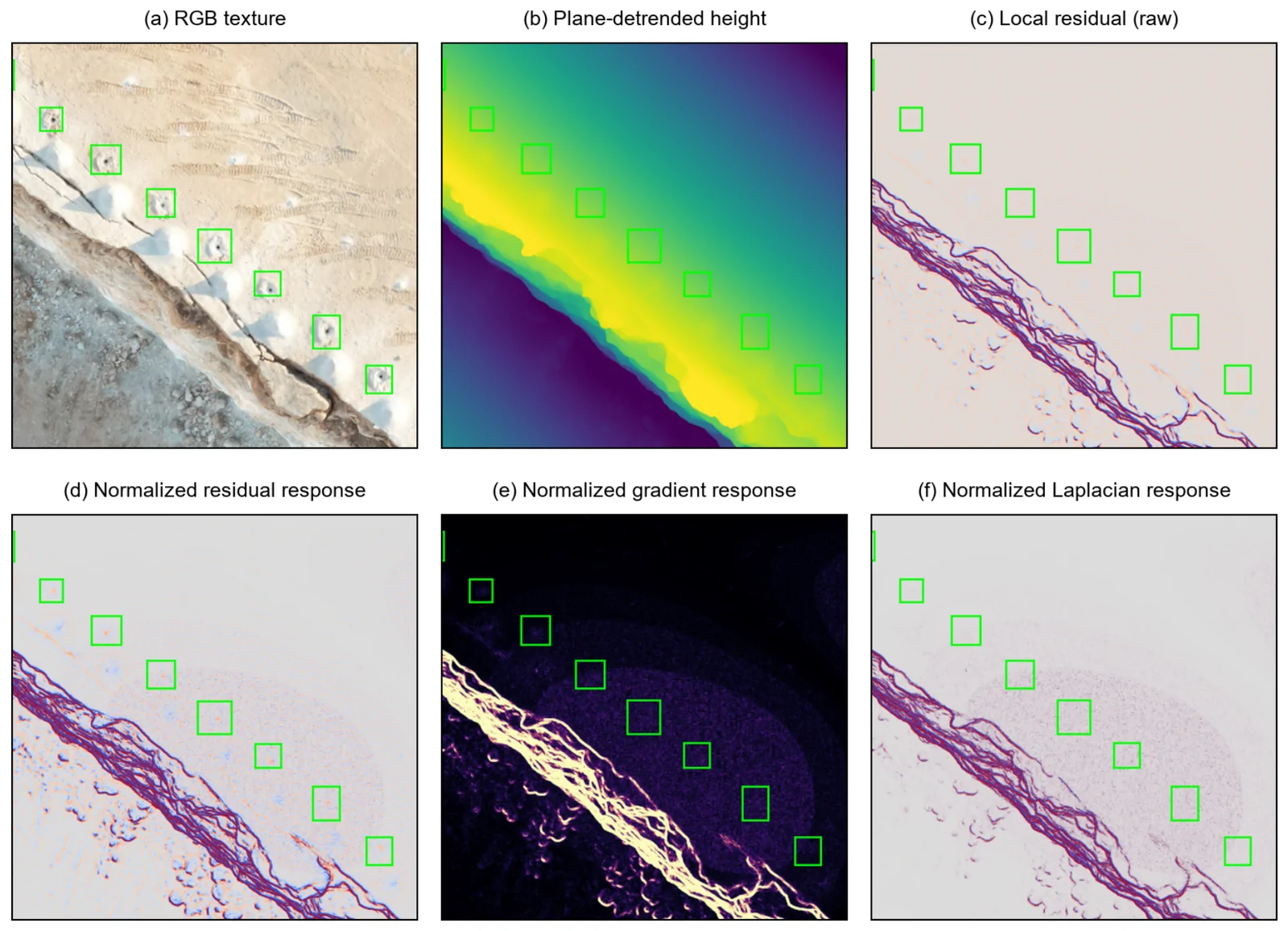
Real RGB and geometry example (S10, patch 000023; $k_{s}=5$). Eight archived patch-local GT boxes, including one narrow box clipped at the left boundary, are drawn in green. Panels (**a**–**c**) show RGB texture, plane-detrended height, and raw residual; (**d**–**f**) show the normalized continuous channels. Each scalar panel uses its own valid-pixel 2nd–98th percentile display limits. Colors show spatial patterns within a panel and do not provide a common amplitude scale across panels. The support mask is uniformly one in this example.

The gradient responds at the flanks of a local depression, while the Laplacian is negative near the maximum of the positive residual and positive at surrounding transitions. Because these operators are evaluated in pixel coordinates, we refer to them as discrete morphology responses rather than metric slope and curvature. Figure 5 shows an aligned real-data example before and after this transform.

**Figure 6:**
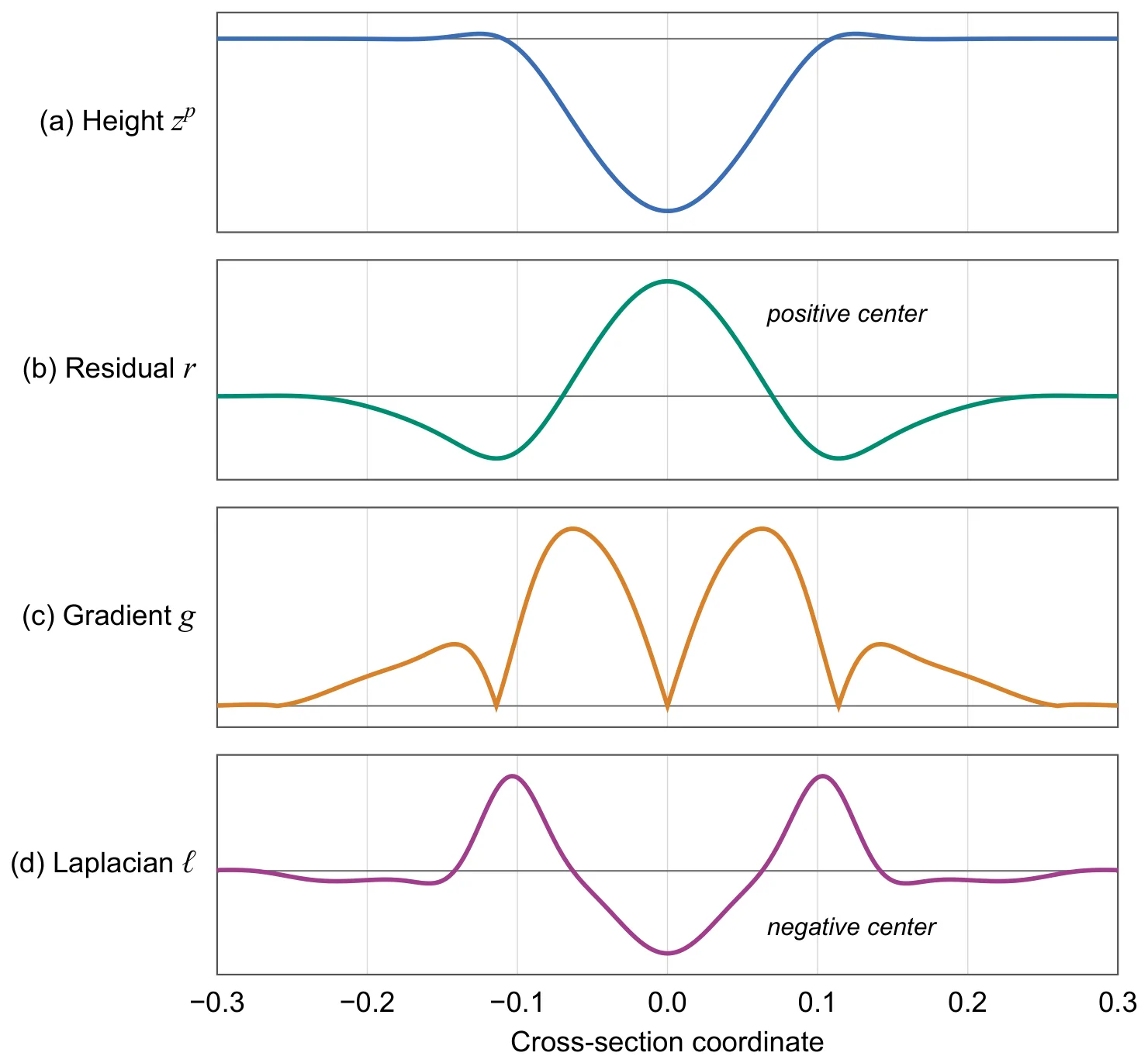
One-dimensional schematic of the geometry transform: (**a**) plane-detrended height $z^{p}$ (blue), (**b**) residual $r=B_{k_{s}}\left(z^{p}\right)-z^{p}$ (green), (**c**) gradient magnitude *g* (orange), and (**d**) Laplacian *ℓ* (purple). A depression produces a positive residual, flank gradient responses, and a negative central second difference. Coordinates and height use arbitrary units; (**b**–**d**) are independently scaled to unit maximum absolute amplitude for display, separately from detector median/IQR normalization.

Figure 6 explains the response signs for an idealized depression. The real example in Figure 5 also contains strong gradient and Laplacian responses along the rock-face boundary outside the annotated targets. Thus, these inputs encode surrounding surface structure as well as aperture-scale variation. RGB supplies appearance and context for distinguishing boreholes from other geometric discontinuities.

The exported height raster can contain unsupported pixels. We retain the accompanying binary support mask $v_{ij}\in \left\{0,1\right\}$ and compute normalization statistics only over Ω.

Because raster derivatives can contain extreme responses, each continuous channel c∈{r,g,l} is standardized per patch with the valid-pixel median and interquartile range (IQR):

(8) $$m_{c}=Q_{0.50,\Omega }\left(c\right),\;s_{c}=Q_{0.75,\Omega }\left(c\right)-Q_{0.25,\Omega }\left(c\right),$$

where $Q_{\alpha ,\Omega }\left(c\right)$ denotes the α-quantile over valid pixels. We normalize and clip as

(9) $${\bar{c}}_{ij}=\mathrm{clip}\;\left(\frac{c_{ij}-m_{c}}{s_{c}+10^{-6}},-10,10\right).$$

After *r* is computed, unsupported residual pixels are filled with the supported-pixel median for finite differences. The one-pixel border of the raw gradient and Laplacian is set to zero. The gradient and Laplacian are multiplied by *v* before valid-pixel normalization, and all unsupported normalized outputs are zeroed. Plane fitting uses supported samples only; diffusion filling supplies values for background filtering, and median filling supplies values for the derivative stencil.

The final geometry encoding is

(10) $$X_{G}=\psi \left(G,v\right)=\left[\bar{r},\bar{g},\bar{l},v\right]\in R^{H\times W\times 4},$$

which is concatenated with RGB in Section 3.6.

### 3.6. Seven-Channel Early Fusion and Stem Initialization

The reported implementation performs input-level fusion. For a sample-level geometry mask m∈{0,1}, the detector input is

(11) $$U_{m}={\mathrm{concat}}_{\mathrm{ch}}\left(I,mX_{G}\right)\in R^{H\times W\times 7}.$$

This preserves pixel correspondence and permits the same detector configuration to be used after adapting only its first convolution. For DINO-R50, let $W^{\left(3\right)}$ be the ImageNet-pretrained three-channel kernel. The seven-channel kernel is initialized as

(12) $$W_{o,c}^{\left(7\right)}=\left\{\begin{array}{cc} W_{o,c}^{\left(3\right)}, & c\in \{1,2,3\},\\ \frac{1}{3}\sum_{q=1}^{3}W_{o,q}^{\left(3\right)}, & c\in \{4,5,6,7\}. \end{array}\right.$$

All stem weights are subsequently fine-tuned. The same channel-expansion rule is applied to the pretrained RGB stem of YOLO11x. For RGB-plus-subset ablations containing q<4 geometry channels, the same rule produces a (3+q)-channel stem: the RGB kernels retain their pretrained values and every additional kernel receives the RGB-kernel mean in Equation (12). The four-channel geometry-only stem initializes each input kernel with that same mean. No separate learned geometry encoder or feature-wise modulation block is used; ψ denotes deterministic raster preprocessing, and the detector neck, head, and loss remain those of the selected baseline.

### 3.7. Joint Geometry Dropout

Geometry may be unavailable at deployment even when it was available during training. Following whole-modality dropout [20], we draw one mask per training sample,

(13) m∼Bernoulli(1−p),

and multiply all four geometry channels, including support, by the same *m*. The objective is

(14) $$\min_{\omega }\;E_{(I,G,v,Y)∼D}\;E_{m}\left[L\left(D_{\omega }\left(\left[I;mX_{G}\right]\right),Y\right)\right].$$

At test time, m=1 denotes geometry-present evaluation and m=0 denotes the all-zero-geometry mode of the same seven-channel model. For p>0, both modes occur during training; for p=0, all-zero geometry is an out-of-distribution stress test. The all-zero mode uses RGB filters jointly trained with geometry-present samples, whereas the RGB baseline has separately trained weights. We evaluate p∈{0,0.1,0.3,0.5} to characterize performance under complete geometry availability and complete tensor removal.

### 3.8. Experimental Design and Implementation

The primary detector is DINO with a ResNet-50 backbone [14,23]. YOLO11x [22] provides a one-stage comparison using the same scene folds, 1024×1024 patches, and 50-epoch training budget. The seven-channel YOLO11x stem uses the initialization in Equation (12). Deformable DETR, Conditional DETR, and Cascade R-CNN provide additional controlled checks across transformer and two-stage families [13,24,25]. For each detector, modality comparisons retain the same neck, head, training budget, and test-time post-processing; only the input representation and corresponding first-layer width change. GeoBoreNet denotes the complete RGB-plus-derived-geometry configuration of the selected detector.

The primary comparison pairs a separately trained three-channel RGB detector with seven-channel GeoBoreNet, $\left[I,\bar{r},\bar{g},\bar{l},v\right]$. The support-only condition has four input channels, [I,v]. The no-support-input condition has six, $\left[I,\bar{r},\bar{g},\bar{l}\right]$: it omits *v* from the network input while retaining *v* for valid-pixel preprocessing and normalization. Comparing this condition with the complete input measures the change when support is supplied as an additional channel. Each ablation evaluates an input configuration together with its corresponding first-layer width. Local-background sensitivity is evaluated at nominal spans of 0.15, 0.30, and 0.60 m, each converted with Equation (5). Geometry-only and local-residualization controls examine the contribution of RGB and the local height reference.

DINO-R50 uses MMDetection [26] and pretrained ResNet-50 weights. RGB intensities are normalized with channel means (123.675,116.28,103.53) and standard deviations (58.395,57.12,57.375) on the 0–255 scale; continuous geometry uses the valid-pixel normalization in Section 3.5. Paired RGB/geometry runs use the same initialization source and optimization schedule. The DINO-R50 configuration trains for 50 epochs with batch size 4. Linear warm-up spans epochs 0–5, followed by cosine annealing through epoch 50 to a minimum learning rate of $10^{-6}$. AdamW uses learning rate $10^{-4}$, weight decay $10^{-4}$, gradient-norm clipping at 0.1, and a 0.1× backbone learning-rate multiplier [27]. Spatial augmentation applies horizontal and vertical flips (probability 0.5 each) and random affine transforms (rotation up to $90^{\circ }$, translation up to 0.1 of image extent, and scale 0.9–1.1) consistently to RGB, continuous geometry, support, and boxes. Each box is transformed through its four corners, converted to the enclosing axis-aligned rectangle, clipped to the image boundary, and removed if degenerate; RGB and continuous geometry use bilinear interpolation and support uses nearest-neighbor interpolation. Checkpoints are selected by validation COCO AP on the validation scene. The primary DINO comparison uses five paired seeds: 1337, 2024, 7, 42, and 3407. Paired models use the same training, validation, and test scenes for each fold and seed.

### 3.9. Evaluation Protocol and Metrics

Bounding-box average precision follows the COCO protocol [28]: AP is averaged over intersection-over-union thresholds 0.50:0.05:0.95, with AP_50_ and AP_75_ also reported. For seed *k*, scene-macro AP is $A_{k}=\frac{1}{11}\sum_{s=1}^{11}{\mathrm{AP}}_{s,k}$, giving each held-out scene equal weight irrespective of its patch count. We report the mean and sample standard deviation of $A_{k}$ over the five paired seeds. Patch-level evaluation retains all observations in overlapping crops; source-object aggregation below evaluates repeated observations at the level of the original annotated target. Paired differences are computed within each held-out scene and seed. Secondary pooled cross-fold AP combines held-out predictions and annotations under unique image identifiers into one evaluation. Scene-macro AP averages the separately evaluated scenes, whereas pooled AP ranks detections across the combined folds.

For source-object evaluation, patch predictions are mapped back to source coordinates, merged across overlapping patches with class-wise non-maximum suppression, and matched to each eligible source rectangle once. We report the resulting source-object-unique AP and an NMS-IoU sensitivity sweep from 0.3 to 0.7. Operational evaluation uses annotation-complete search regions containing both positive and zero-object tiles. It reports AP, AP_50_, precision at fixed recall levels, and false positives per 100 tiles. Here, an annotation-complete region means that all visible targets within its evaluation boundary are included in the ground truth. Performance is measured within those boundaries.

Precision–recall (PR) curves summarize detection performance across confidence thresholds. Precision is the fraction of predicted boxes matched to GT, and recall is the fraction of eligible GT targets detected. The operational evaluation compares RGB and RGB+Derived within each detector family using the same operational regions and GT denominator, with a matching IoU threshold of 0.50. AP_50_ is the mean interpolated precision over 101 recall levels from 0 to 1 in steps of 0.01. Precision at recall 0.5 and 0.7 is read from this curve. At confidence threshold τ, false positives per 100 tiles are $100N_{\mathrm{FP}}\left(\tau \right)/\left(N_{+}+N_{0}\right)$, where $N_{+}$ and $N_{0}$ are the numbers of positive and zero-object tiles. All evaluated tiles contribute to this denominator. AP and the PR curve are computed across confidence thresholds.

## 4. Results

We first report the five-seed RGB–geometry comparison, followed by a scene-wise breakdown, representation and scale studies, detector-family comparisons, source-object aggregation, operational evaluation with background tiles, qualitative examples, and geometry-availability analysis.

### 4.1. Cross-Scene RGB–Geometry Comparison

Across the five paired seeds, the scene-macro AP difference ranges from 0.063 to 0.065 and is positive in every replicate (Table 4). Table 5 provides the scene-level breakdown for seed 1337. The seed-level and scene-level tables summarize training variation and variation among held-out scenes, respectively.

For seed 1337, the paired change is positive in all 11 held-out scenes and ranges from 0.034 to 0.095 AP. The scene-macro AP changes from 0.481±0.033 to 0.546±0.029, with a scene-wise paired mean change of 0.064±0.015 AP. These scene-wise values quantify directional consistency but remain descriptive: the acquisition record does not establish independent physical sampling units. Table 4 uses the displayed seed-level means to compute differences; here the differences are averaged from the displayed scene-level values. Rounding at these different levels gives 0.065 and 0.064, respectively, for seed 1337. The five-seed summary characterizes stochastic training variation.

### 4.2. Representation Component Analysis

Shadows and dark rocks can resemble boreholes in RGB, whereas a physical depression produces a positive local-background residual under Equation (4). Geometry-only results show that not every depression is a borehole; the value of the encoding is therefore assessed through its incremental contribution when paired with RGB.

#### 4.2.1. Geometry-Channel Contributions

Table 6 compares individual and combined geometry responses added to RGB, including controls with support alone and with all three continuous responses but no support input. Among the single-continuous-response conditions, all of which also include *v*, the residual-plus-support input has the highest AP (0.502). The support-only condition reaches 0.498 AP, whereas RGB with all three continuous responses but no support input reaches 0.538 AP. Adding support to those three responses changes AP from 0.538 to 0.548. The six-channel continuous-response input outperforms both RGB and RGB plus support, and the complete seven-channel input has the highest AP among the tested conditions.

#### 4.2.2. Effect of Local Residualization

Table 7 compares morphology channels computed directly from the plane-detrended field $z^{p}$ with channels computed from the local box-filter residual *r*. For the first row, $z^{p}$, $g(z^{p})$, and $l(z^{p})$ receive the same valid-pixel median/IQR standardization and clipping defined in Equations (8) and (9). The locally residualized representation changes secondary pooled AP from 0.521 to 0.548 (5.2% relative), consistent with a benefit from referencing height to the local background rather than the fitted patch-wide plane.

#### 4.2.3. Local-Background Scale Sensitivity

The reference span specifies the neighborhood over which height is compared with its background. We assess dependence on this choice using nominal spans of 0.15, 0.30, and 0.60 m. Each is converted with the scene-specific metadata resolution in Equation (5), giving the pixel-window ranges in Table 8.

The AP range across the three settings is 0.013. The 0.30 m reference has the highest pooled AP (0.548), compared with 0.541 and 0.535 for the smaller and larger settings. These results characterize sensitivity over the tested neighborhoods. Their conversion to physical distances depends on the mesh-coordinate units used in the resolution metadata.

### 4.3. Consistency Across Detector Families

Table 9 compares input configurations within each detector family. Adding the complete derived representation increases pooled AP by 0.065 for DINO, 0.060 for Deformable DETR, 0.058 for Conditional DETR, 0.070 for Cascade R-CNN, and 0.065 for YOLO11x. Geometry therefore remains useful in the one-stage YOLO11x comparison as well as the transformer and two-stage comparisons. Because training recipes differ across detector families, we interpret the geometry gain within each family.

### 4.4. Source-Object-Unique Evaluation

The target-centered construction causes the same source rectangle to appear in several overlapping patches. To prevent frequently repeated targets from receiving greater evaluation weight, predictions are transformed back to source coordinates, merged, and matched to each source rectangle once. Table 10 reports the unique-object AP and the sensitivity of that result to the cross-patch NMS threshold.

At NMS IoU 0.4, source-object-unique AP is 0.448 for RGB and 0.493 for RGB+Derived, a difference of 0.045. Across NMS IoU 0.3–0.7, the AP ranges are 0.005 and 0.006, respectively, and the paired ordering is unchanged. The unique-object result therefore retains a geometry benefit after repeated predictions of the same annotated source target are merged.

### 4.5. Operational Evaluation with Negative Background Tiles

Target-centered patches condition evaluation on known target presence. The operational comparison instead evaluates sliding-window tiles from annotation-complete regions, including tiles with no ground-truth boreholes. AP summarizes detections across confidence thresholds, while FP/100 tiles measure the false-positive burden at the recorded operating point. The result applies to the evaluated regions.

As shown in Table 11, adding the derived representation changes DINO-R50 operational AP from 0.38 to 0.43 and reduces the observed false positives from 8.0 to 4.0 per 100 tiles. The paired YOLO11x comparison changes AP from 0.30 to 0.36 and false positives from 9.5 to 6.0 per 100 tiles. These results extend the representation comparison beyond regions selected solely because a target was already known to be present.

### 4.6. Qualitative Detection Analysis

**Figure 7:**
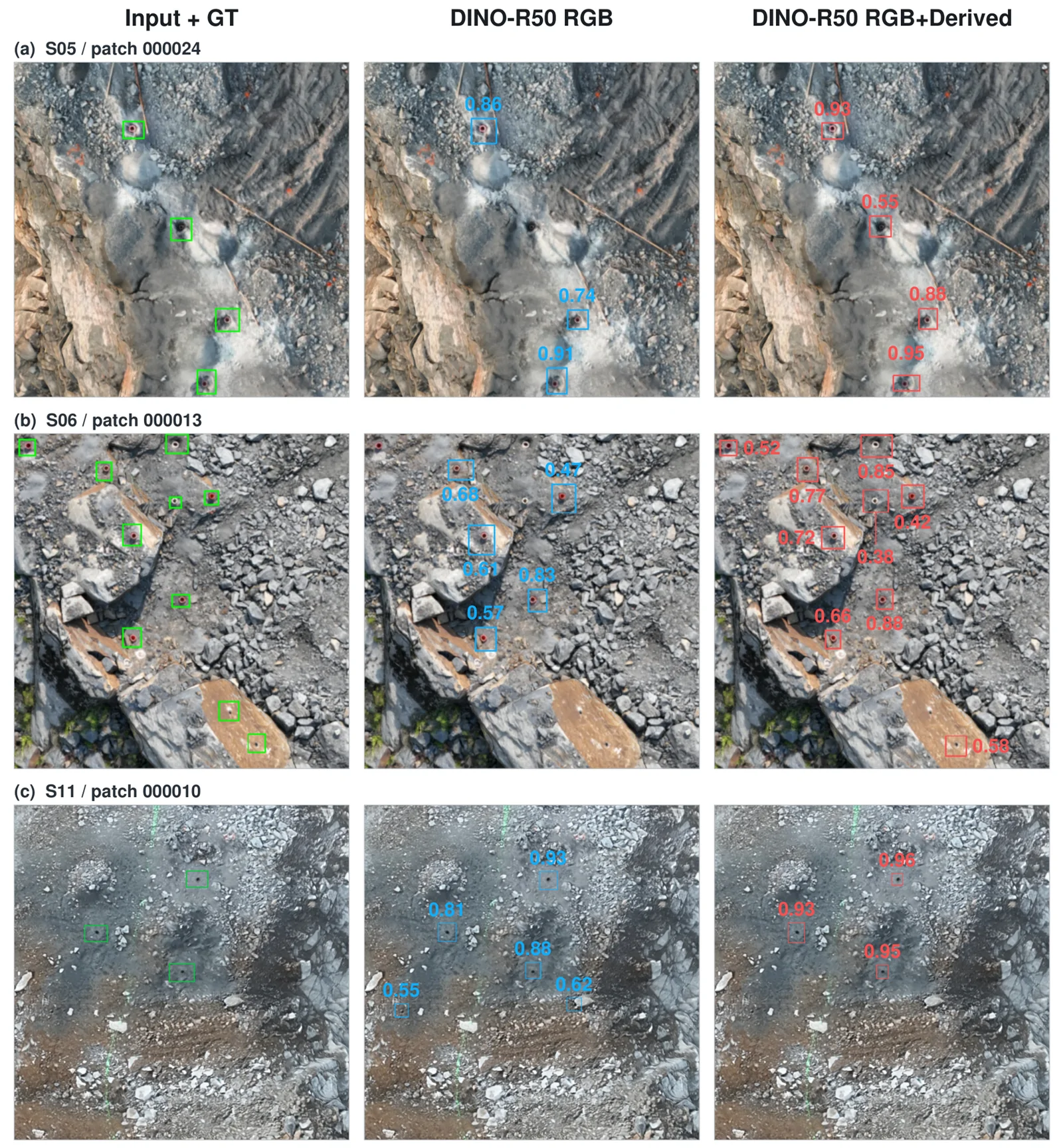
Qualitative DINO-R50 detections: (**a**) S05 patch 000024; (**b**) S06 patch 000013; (**c**) S11 patch 000010. Columns show input/GT (green), RGB predictions (blue), and RGB+Derived predictions (red). Numbers indicate prediction confidence scores. The same patches and GT are used in Figure 8.

**Figure 8:**
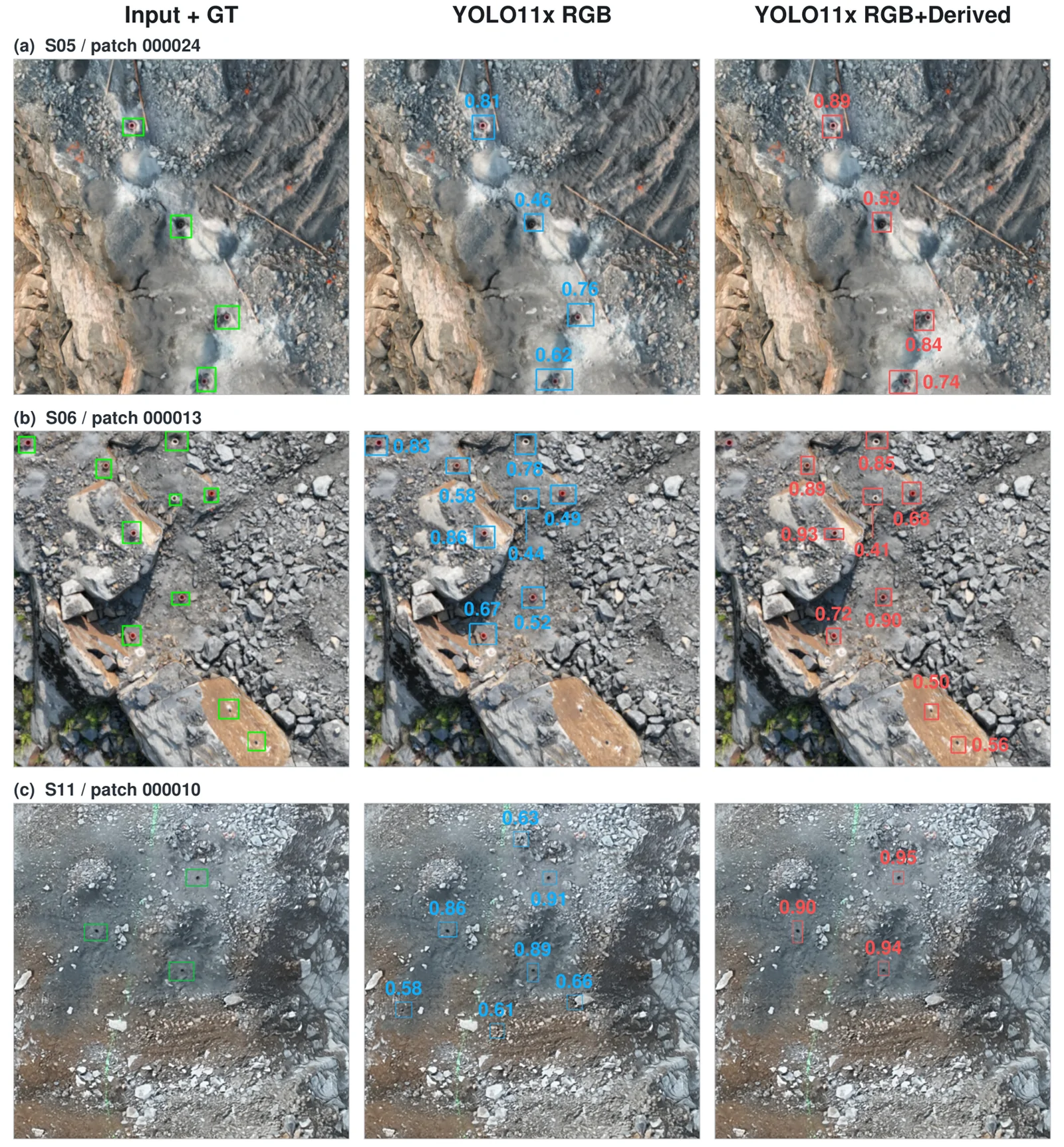
Qualitative YOLO11x detections on the same patches as Figure 7, with the same column layout and colors. Numbers indicate prediction confidence scores.

Figure 7 and Figure 8 compare DINO-R50 and YOLO11x on S05 patch 000024, S06 patch 000013, and S11 patch 000010, containing four, ten, and three GT boxes, respectively. Each patch has complete reconstruction support. Rows retain the same image content and GT across detector families. Green GT boxes appear only in the first column; the other columns show RGB predictions (blue) and RGB+Derived predictions (red). Each predicted box carries its confidence score to two decimal places.

For the qualitative comparison, displayed predictions are matched to GT one-to-one in descending confidence order at IoU ≥0.50. Matched predictions are true positives (TP); unmatched predictions and GT are false positives (FP) and false negatives (FN), respectively.

The upper opening in S05 provides a true-positive example for both input settings and detector families. In S06, geometry enables detection of additional small openings, while DINO-R50 still misses the upper opening on the lower orange rock. In S11, RGB produces background false alarms among dark stones and gaps that are absent from the RGB + Derived panels. Some tighter predictions around actual openings nevertheless fail the IoU criterion, illustrating localization error alongside background confusion. These cases complement the aggregate detection and background-search metrics in Table 11.

**Figure 9:**
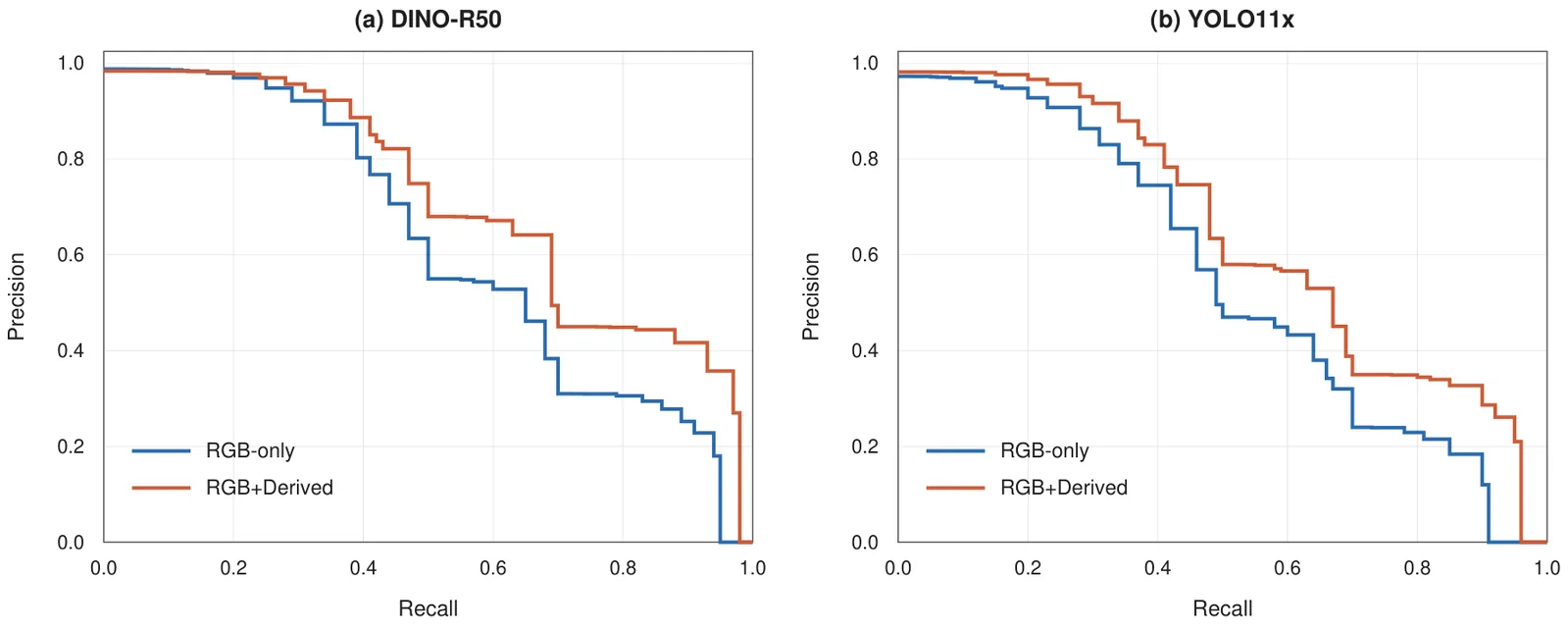
Operational precision–recall curves at IoU 0.50 for (**a**) DINO-R50 and (**b**) YOLO11x. Each curve uses 101 recall samples; Table 11 gives the corresponding metrics.

Figure 9 shows the corresponding PR curves for DINO-R50 and YOLO11x. RGB+Derived maintains higher precision over most of the recall range for both detectors. DINO-R50 AP_50_ increases from 0.62 to 0.71, and YOLO11x AP_50_ increases from 0.55 to 0.64. At recall 0.70, precision increases from 0.31 to 0.45 for DINO-R50 and from 0.24 to 0.35 for YOLO11x, consistent with Table 11.

### 4.7. Geometry-Availability Trade-Off

For each training dropout rate *p*, the same fold-specific DINO-R50 checkpoint is evaluated twice: once with geometry present and once with all four geometry inputs zeroed. Different values of *p* correspond to separately trained models. Every model retains seven input channels in both test modes.

Table 12 shows a consistent trade-off between performance with geometry present and performance with geometry absent. Increasing *p* raises all-zero-geometry AP from 0.487 to 0.503 while changing geometry-present AP from 0.548 to 0.520. At p=0.3, the same fold-specific weights yield 0.532 AP with geometry and 0.496 with geometry zeroed; at p=0.5, the relative gap narrows to 3.3%. Each row compares two input conditions for the same trained seven-channel model; Table 9 reports the separately trained three-channel RGB baseline.

## 5. Discussion

The five paired seeds yield DINO-R50 scene-macro AP of 0.4808±0.0059 for RGB and 0.5446±0.0059 for GeoBoreNet. All paired seed differences are positive, and geometry improves AP in all 11 held-out scenes for seed 1337. Seed-level SD describes variation from stochastic training, while scene-level SD describes variation among the evaluated sources.

The component results identify useful input configurations. RGB plus support reaches 0.498 pooled AP, the six-channel RGB-plus-continuous-response input reaches 0.538, and the complete seven-channel input reaches 0.548. The continuous-response configuration therefore performs better than the support-only input. All conditions still use valid-pixel preprocessing, and changing channel count changes the first-layer parameter count; the measured gains reflect both the supplied channels and the associated change in first-layer width. In Figure 5, geometry responses also highlight the rock-face edge. These background structures illustrate ambiguity in geometry-only inputs, whose AP values range from 0.085 to 0.132 in Table 9. RGB adds appearance and context to the same local geometry.

Local-background subtraction changes pooled AP from 0.521 to 0.548 relative to plane detrending alone. Nominal background spans of 0.15, 0.30, and 0.60 m give 0.541, 0.548, and 0.535 AP. The benefit is therefore present across the tested neighborhood sizes. Physical interpretation of these spans requires the coordinate units underlying the recorded raster resolution; detector-grid target widths and heights describe image sampling, not aperture diameters.

The complete representation improves AP within all five detector families. YOLO11x AP increases from 0.385 to 0.450, showing that local geometry can complement a one-stage RGB detector. This paired result addresses a different question from ranking detector architectures across their training recipes. Prior UAV-photogrammetric studies address broad-area detection [5], while RGB-D, LiDAR, and binocular systems often target close-range localization [3,8,11]. GeoBoreNet uses an available textured surface model to supply aligned morphology channels for two-dimensional box detection.

Source-object merging and background search address two separate effects of target-centered patches. At cross-patch NMS IoU 0.4, source-object-unique AP rises from 0.448 to 0.493; the paired ordering holds throughout the 0.3–0.7 sweep. In the operational regions, DINO-R50 AP increases from 0.38 to 0.43 and YOLO11x AP from 0.30 to 0.36. FP/100-tile values decrease from 8.0 to 4.0 and from 9.5 to 6.0, respectively. AP measures performance across confidence thresholds, while FP/100 tiles quantifies false positives at the selected operating points. Figure 7 and Figure 8 illustrate additional detections and changes in box placement, and Figure 9 shows the improved precision–recall balance with geometry.

Geometry dropout improves performance when the geometry tensor is absent, at the cost of performance when geometry is present. Increasing *p* from 0 to 0.5 changes present-geometry AP from 0.548 to 0.520 and zero-geometry AP from 0.487 to 0.503. Within each row, both values use the same seven-channel model. The separately trained RGB baseline remains a distinct model.

Evaluation uses 11 source scenes and two-dimensional boxes around visible openings; acquisition records do not establish independent physical quarry sites. Operational results cover the annotated evaluation regions. Additional sites, partial surface support, geometry corruption, and alignment perturbations would extend the assessment of reconstruction quality and detection performance.

## 6. Conclusions

GeoBoreNet introduces local residual, spatial-gradient, spatial-Laplacian, and support channels alongside RGB through an expanded first convolution. Five-seed scene-disjoint evaluation gives a mean paired DINO-R50 AP increase of 0.0638. Component, scale, detector-family, source-object-unique, and background-tile comparisons favor the complete representation in the reported data. Geometry dropout improves robustness to missing geometry at the cost of complete-input accuracy. These results support local surface morphology as a complementary input for two-dimensional borehole detection.

## Figures and Tables

**Table 1:** DINO-R50 GeoBoreNet architecture for a 1024×1024 input. Here, $n^{2}$ denotes an n×n spatial map; channel counts follow the multiplication sign. BN, GN, and FFN denote batch normalization, group normalization, and feed-forward network, respectively.

Block	Operation	Input Shape	Output Shape	Notes
Input	channel concatenation	$1024^{2}\times \left(3+4\right)$	$1024^{2}\times 7$	RGB normalization; robust geometry normalization
Stem	7×7 conv, stride 2	$1024^{2}\times 7$	$512^{2}\times 64$	BN + ReLU
Pool/C2	max-pool + ResNet stage	$512^{2}\times 64$	$256^{2}\times 256$	BN + ReLU, residual blocks
C3	ResNet stage, stride 2	$256^{2}\times 256$	$128^{2}\times 512$	BN + ReLU, residual blocks
C4	ResNet stage, stride 2	$128^{2}\times 512$	$64^{2}\times 1024$	BN + ReLU, residual blocks
C5	ResNet stage, stride 2	$64^{2}\times 1024$	$32^{2}\times 2048$	BN + ReLU, residual blocks
Projection	four-scale channel mapper	C3/C4/C5	$128^{2},64^{2},32^{2},16^{2}$, each 256 ch.	GN
Encoder	6 deformable-attention layers	four 256-ch. scales	encoded multiscale tokens	8-head attention + 2048-d FFN
Decoder	6 query-decoder layers	encoded tokens + 900 queries	900×256	self/cross-attention + FFN
Class head	linear prediction	900×256	900×1	focal classification loss, weight 1
Box head	iterative MLP prediction	900×256	900×4	L1 loss, weight 5; GIoU loss, weight 2

**Table 2:** Composition of the aligned source data and detector corpus.

Item	Value
Annotated source scenes	11
Native source texture dimensions	Scene-specific; archived with source inventory
Texture-coordinate annotation rectangles	683
Successfully projected detector targets	675
Annotations without a detector-grid projection	8
Target-centered detector patches	675 at 1024×1024 pixels
Positive/negative patches	675/0
Evaluated patch-level box records	6374 (repeated appearances included)
Detection class	1 (borehole)

**Table 3:** Per-scene metadata resolution, target count, patch density, ground-truth (GT) target scale, and nominal 0.3 m preprocessing window. Source/mapped GT gives the number of texture-coordinate source rectangles and successfully mapped unique GT targets. Intersections/patch gives the mean, median, and range of positive-area mapped rectangles intersecting each constructed patch, before exclusion of the seven boundary slivers during detector-manifest conversion. GT width, height, and area are separate medians over unique mapped rectangles on the detector grid; consequently, median width multiplied by median height need not equal median area. These pixel-space statistics characterize detector sampling and are not estimates of physical aperture diameter.

Scene	Metadata $\rho_{s}$ (m px^−1^)	Source/ Mapped GT	Patches	Intersections/Patch Mean; Med.; Range	GT Width (px)	GT Height (px)	GT Area (px^2^)	$k_{s}$ (px)
S01	0.01977	66/66	66	16.88; 17; 8–24	56	59	3290	15
S02	0.00500	133/133	133	1.88; 2; 1–5	117	122	14,490	61
S03	0.07331	35/35	35	14.86; 15; 9–21	49	47	2352	5
S04	0.11474	26/26	26	8.77; 9.5; 3–15	45	45	1956	3
S05	0.00945	26/26	26	3.81; 3.5; 2–6	63	58.5	3713.5	33
S06	0.00953	122/115	115	7.77; 7; 2–15	63	61	3528	31
S07	0.10438	169/169	169	14.27; 13; 5–23	117	113	12,870	3
S08	0.07236	25/25	25	8.12; 8; 5–11	156	143	21,945	5
S09	0.07347	38/37	37	10.57; 11; 4–14	49	49	2352	5
S10	0.06311	25/25	25	7.48; 8; 3–9	76	74	5628	5
S11	0.02957	18/18	18	4.67; 5; 3–6	80	90.5	7298.5	11

**Table 4:** Five-seed DINO-R50 comparison. Each entry is the macro mean over the 11 held-out scenes for one paired seed. Paired differences and the final mean ± sample SD are computed from the displayed seed-level values.

Seed	RGB Macro AP	GeoBoreNet Macro AP	Paired ΔAP
1337	0.481	0.546	+0.065
2024	0.476	0.540	+0.064
7	0.488	0.551	+0.063
42	0.474	0.537	+0.063
3407	0.485	0.549	+0.064
Mean ± SD	0.4808 ± 0.0059	0.5446 ± 0.0059	+0.0638 ± 0.0008

**Table 5:** Held-out-scene DINO-R50 AP for RGB-only and GeoBoreNet for the representative paired seed 1337. ΔAP is paired by source scene, and the macro row reports the mean ± sample standard deviation over scenes.

Source Scene	RGB AP	RGB + Derived AP	ΔAP
Scene 1	0.533	0.599	+0.066
Scene 2	0.481	0.515	+0.034
Scene 3	0.422	0.504	+0.082
Scene 4	0.517	0.568	+0.051
Scene 5	0.448	0.543	+0.095
Scene 6	0.476	0.538	+0.062
Scene 7	0.502	0.564	+0.062
Scene 8	0.463	0.524	+0.061
Scene 9	0.488	0.555	+0.067
Scene 10	0.456	0.522	+0.066
Scene 11	0.508	0.570	+0.062
Macro mean ± SD	0.481 ± 0.033	0.546 ± 0.029	+0.064 ± 0.015

**Table 6:** Secondary pooled cross-fold geometry-channel and support-mask ablation for DINO-R50. RGB is fixed, and the geometry inputs are varied. The channel-count column lists geometry/total channels, including the three RGB channels. No support means that *v* is omitted from the network input; supported-pixel preprocessing is retained. ΔAP is reported in AP points relative to RGB-only.

Geom Input	Notation	Geom/Total	AP	ΔAP (pts)	AP_50_/AP_75_
RGB only	–	0/3	0.483	–	0.714/0.557
Support only	[v]	1/4	0.498	+1.5	0.725/0.568
$\bar{r}$	$\left[\bar{r},v\right]$	2/5	0.502	+1.9	0.736/0.579
$\bar{g}$	$\left[\bar{g},v\right]$	2/5	0.497	+1.4	0.730/0.573
$\bar{l}$	$\left[\bar{l},v\right]$	2/5	0.494	+1.1	0.724/0.566
$\bar{r}+\bar{g}$	$\left[\bar{r},\bar{g},v\right]$	3/6	0.527	+4.4	0.759/0.605
$\bar{r}+\bar{l}$	$\left[\bar{r},\bar{l},v\right]$	3/6	0.516	+3.3	0.748/0.591
$\bar{r}+\bar{g}+\bar{l}$ (no *v* input)	$\left[\bar{r},\bar{g},\bar{l}\right]$	3/6	0.538	+5.5	0.772/0.618
Full (GeoBoreNet)	$\left[\bar{r},\bar{g},\bar{l},v\right]$	4/7	**0.548**	**+6.5**	**0.783/0.628**

**Table 7:** Effect of local-background residualization on secondary pooled cross-fold AP after planar detrending (DINO-R50, RGB+Geom). The first row derives responses directly from $z^{p}$; the second uses $r=B_{k_{s}}\left(z^{p}\right)-z^{p}$. Other settings are fixed, and geometry is present at test time.

Base Height	Derived Representation (with RGB)	AP	AP_50_	AP_75_
$z^{p}$ (plane detrended)	$\left[{\bar{z}}^{p},\;\bar{g(z^{p})},\;\bar{l(z^{p})},\;v\right]$	0.521	0.738	0.603
*r* (local residual)	$\left[\bar{r},\;\bar{g},\;\bar{l},\;v\right]$	**0.548**	**0.783**	**0.628**

**Table 8:** Sensitivity of DINO-R50 with RGB plus the complete derived representation to the nominal local-background span defined by the metadata resolution. Values are secondary pooled cross-fold metrics.

Nominal Span	Scene-Specific $k_{s}$ Range	AP	AP_50_	AP_75_	ΔAP vs. 0.30 m
0.15 m	3–31	0.541	0.776	0.622	−0.007
0.30 m	3–61	0.548	0.783	0.628	–
0.60 m	5–121	0.535	0.768	0.614	−0.013

**Table 9:** Detector-family comparison under the same scene-disjoint protocol. *Derived* denotes $\left[\bar{r},\bar{g},\bar{l},v\right]$. Multimodal rows use geometry at test time and no geometry dropout (p=0); RGB-only rows are separate three-channel models. Values are secondary pooled cross-fold metrics; the primary multi-seed scene-macro comparison is reported in Table 4.

Model	Train Input	Geom Rep	AP	AP_50_	AP_75_
DINO-R50	RGB only	–	0.483	0.714	0.557
	Geom only	Derived	0.132	0.238	0.143
	RGB + Geom	Derived	**0.548**	**0.783**	**0.628**
Deformable DETR-R50	RGB only	–	0.463	0.697	0.521
	Geom only	Derived	0.127	0.215	0.124
	RGB + Geom	Derived	**0.523**	**0.764**	**0.605**
Conditional DETR-R50	RGB only	–	0.443	0.678	0.509
	Geom only	Derived	0.108	0.192	0.107
	RGB + Geom	Derived	**0.501**	**0.746**	**0.587**
Cascade R-CNN-R50	RGB only	–	0.416	0.654	0.457
	Geom only	Derived	0.085	0.163	0.081
	RGB + Geom	Derived	**0.486**	**0.713**	**0.539**
YOLO11x	RGB only	–	0.385	0.620	0.420
	RGB + Geom	Derived	**0.450**	**0.700**	**0.500**

**Table 10:** Source-object-unique DINO-R50 evaluation after mapping predictions back to source coordinates. Columns 0.3–0.7 report AP after cross-patch NMS at the indicated IoU threshold.

Input	NMS 0.3	NMS 0.4	NMS 0.5	NMS 0.6	NMS 0.7
RGB	0.446	0.448	0.449	0.447	0.444
RGB + Derived	0.491	0.493	0.494	0.492	0.488

**Table 11:** Operational evaluation in annotation-complete regions containing positive and zero-object background tiles. Precision is reported at recall 0.5 and 0.7; FP/100 denotes $100N_{\mathrm{FP}}/N_{\mathrm{tiles}}$ at the recorded operating point, with both positive and zero-object tiles in the denominator.

Detector	Input	AP	AP_50_	P@R = 0.5	P@R = 0.7	FP/100 Tiles
DINO-R50	RGB	0.38	0.62	0.55	0.31	8.0
	RGB + Derived	**0.43**	**0.71**	**0.68**	**0.45**	**4.0**
YOLO11x	RGB	0.30	0.55	0.47	0.24	9.5
	RGB + Derived	**0.36**	**0.64**	**0.58**	**0.35**	**6.0**

**Table 12:** Secondary pooled cross-fold geometry-dropout ablation on DINO-R50 with the proposed encoding. Each row is a separately trained seven-channel model. $\Delta \mathrm{AP}={\mathrm{AP}}_{\mathrm{zeroed}}-{\mathrm{AP}}_{\mathrm{present}}$, and degradation is $-\Delta \mathrm{AP}/{\mathrm{AP}}_{\mathrm{present}}$. The p=0 all-zero result is an out-of-distribution stress test; models with p>0 encountered all-zero geometry during training.

Dropout *p*	AP (Present)	AP (All Zero)	ΔAP	Degradation
0.0	0.548	0.487	−0.061	11.1%
0.1	0.544	0.491	−0.053	9.7%
0.3	0.532	0.496	−0.036	6.8%
0.5	0.520	0.503	−0.017	3.3%

## Data Availability

The textured surface meshes, associated RGB textures, and annotations were provided by Austin Powder and are not publicly available because of contractual and source-confidentiality restrictions. Requests for access may be directed to the corresponding author and require written permission from Austin Powder. Derived aggregate metrics reported in the article are included in the tables.
